# North Eastern Iowa Veterinary Medical Association

**Published:** 1892-02

**Authors:** J. L. Kennedy

**Affiliations:** Secretary


*North-Eastern Iowa Veterinary Medical Association.*—The second meeting of the North-Eastern Iowa Veterinary Medical Association was called to order by President Scott, in the parlors of the Turner House, Independence, Ia., January 14, 1892. After roll-call the minutes of the previous meeting were read and approved, Letters of regret were read from Drs. J. W. Brown, V. S.; Oskaloosa, John Tillie, D. V. M., Muscatine, J. E. King, V. S., Auamosa, and W. B. Niles and D. V. M., Ames. The applications of Drs. Wm. Drinkwater, V. S., Monticello, A. S. Barnes, V. S., Maquoketa, and A. S. Brodie, V. S. Cedar Falls, were reported favorably by the Board of Censors and they were elected members.

The subjects: Chronic cough, acute anæmia, influenza, obstetrical operations and actinomycosis were discussed at length. The discussion on actinomycoces was particularly interesting, all of the members expressing their firm belief in the infectious nature of the disease and that animals so afflicted are unfit for human food, and are a source of danger in regard to other animals in which they may come in contact. That it behoves us as veterinarians and preservers of the public health to discountenance the removal of Actinomycotic tumors, thereby obliterating a valuable means of diagnosis and also enabling the owner of such animal to dispose of it to unsuspecting parties. Cases where cited of stockmen who make a business of buying such animals at low prices and employing a veterinarian to remove the tumor—afterwards ship same animals to Chicago (where they sell as first-rate steers) or sell them to local butchers. All of the above cases were proved to be Actinomycotic tumors by a microscopic examination. Dr. Brodie reported some very mysterious cases occurring in horses simulating diptheria, owing to the formation of false membranes in the bronchial tubes. (some of which membranes he exhibited). The President took charge of the specimens, promising to have it examined by an expert microscopist and report the result at next meeting.

A petition to change the name of the Association from North-Eastern to Eastern Iowa Veterinary Medical Association was laid on the table to be acted upon at the next meeting. The Association then adjourned to meet in Cedar Rapids, March 31st, 1892.

J. L. KENNEDY, *Secretary.*

